# Metal halide perovskites for energy applications: recent advances, challenges, and future perspectives

**DOI:** 10.1039/d5ra02730f

**Published:** 2025-06-26

**Authors:** Sonia Soltani, Mokhtar Hjiri, Najwa Idris A. Ahmed, Anouar Jbeli, Abdullah M. Aldukhayel, Nouf Ahmed Althumairi

**Affiliations:** a Department of Physics, College of Science, Qassim University Buraidah 51452 Saudi Arabia S.soltani@qu.edu.sa; b Department of Physics, College of Sciences, Imam Mohammad Ibn Saud Islamic University (IMSIU) Riyadh 11623 Saudi Arabia; c Department of Physics, College of Science, Majmaah University Al-Majmaah 11952 Saudi Arabia

## Abstract

Metal halide perovskites (MHPs) have rapidly emerged as a leading class of materials for a wide range of energy applications, including photovoltaics, light-emitting devices, and energy storage systems. Their exceptional optoelectronic properties such as high absorption coefficients, long carrier diffusion lengths, and tunable bandgaps combined with their low-cost, solution-processable synthesis methods, position MHPs at the forefront of next-generation sustainable energy technologies. Despite these advantages, critical challenges remain, particularly concerning their long-term operational stability, environmental toxicity (especially due to the lead content), and scalability for industrial production. This review comprehensively examines recent progress in the synthesis and characterization of MHPs, focusing on key breakthroughs in materials design, processing techniques, and analytical tools that deepen our understanding of their structure property performance relationships. We also discuss the primary bottlenecks limiting commercial deployment and highlight emerging strategies to improve device durability, reduce ecological impact, and enhance compatibility with scalable manufacturing processes. Finally, we offer a forward-looking perspective on promising research directions aimed at expanding the applicability of MHPs beyond photovoltaics, including their potential roles in thermoelectric conversion, solid-state batteries, and advanced optoelectronic sensors, thereby underscoring their transformative potential in the future of clean energy technologies.

## Abstract 2

Metal halide perovskites (MHPs) have rapidly emerged as a leading class of materials for a wide range of energy applications, including photovoltaics, light-emitting devices, and energy storage systems.

## Graphical abstract

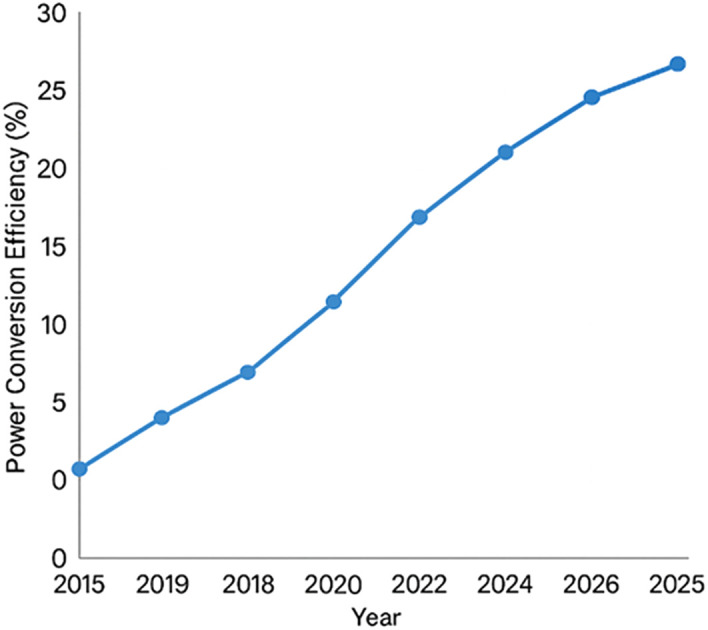

## 1. Introduction

The 21st century has ushered in a critical era for science and engineering, defined by the urgent need to transition toward cleaner, more sustainable energy systems. Rapid urbanization, climate instability, resource depletion, and escalating energy demand have compelled governments, industries, and research communities to reimagine the future of energy production, storage, and consumption.^1–22^ As conventional fossil fuels continue to dominate the global energy mix contributing to greenhouse gas emissions and environmental degradation the importance of renewable technologies such as solar, wind, and thermal energy has grown exponentially.^23–37^ Yet, these technologies alone are not sufficient; they require integration with advanced materials and storage systems to ensure reliability, scalability, and economic viability.^38–51^ The materials science community has responded by exploring a vast array of functional materials that offer high performance, low cost, and environmental sustainability for next-generation energy devices.^52–64^ Innovations in nanomaterials, semiconductors, ionic conductors, and hybrid composites have created new frontiers for energy conversion and storage.^65–78^ Among these, semiconductors with tenable electronic structures have played a central role in driving progress in photovoltaics, thermoelectric, and photodetection.^79–92^ Breakthroughs in materials engineering, especially at the interface of organic–inorganic hybrid materials, have shown that control over atomic composition, dimensionality, and crystallinity can drastically alter device efficiency and stability.^93–105^ In parallel, computational modelling, machine learning, and high-throughput screening have enabled accelerated discovery and optimization of materials tailored for energy applications.^106–122^ Efforts to engineer cost-effective and scalable materials are increasingly supported by global policies focused on circular economy principles, reduced carbon footprints, and environmentally benign alternatives to rare or toxic elements.^123–135^ Moreover, cross-disciplinary integration of materials science with chemistry, electronics, photo physics, and environmental science has become essential for addressing the complex challenges of real-world energy systems.^136–145^ In recent years, researchers have increasingly emphasized the importance of multifunctional materials those capable of serving dual roles in energy generation, storage, or sensing thus enabling the development of compact, integrated energy platforms.^146–153^

**Fig. 1:**
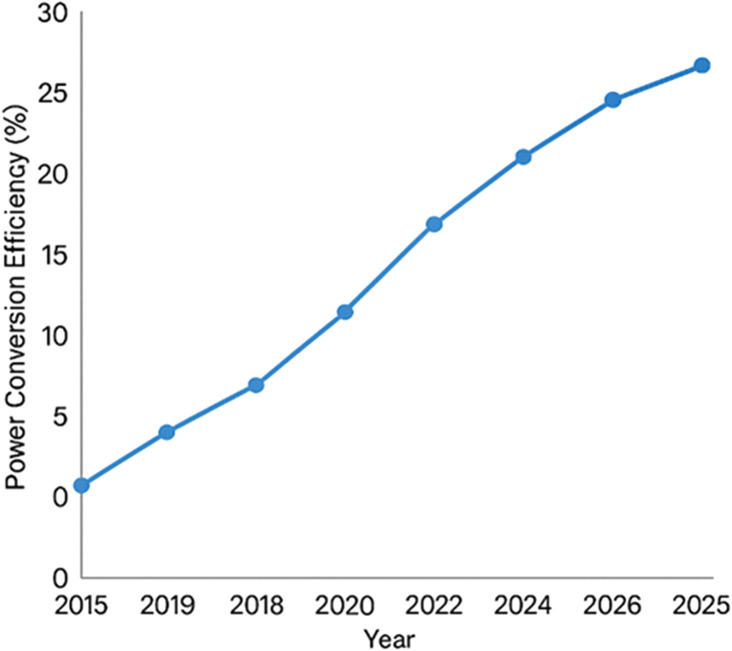
Advancements in power conversion efficiency of metal halide perovskite solar cells (2015–2025).

In this environment of innovation and necessity, metal halide perovskites have emerged as one of the most versatile and high-performing families of materials with transformative implications for energy technologies.^154–156^ With a general chemical formula of ABX_3_, MHPs consist of a monovalent cation (A = methylammonium CH_3_NH_3_^+^, formamidinium HC(NH_2_)_2_^+^, or cesium Cs^+^), a divalent metal cation (B=Pb^2+^, Sn^2+^), and a halide anion (X = Cl^−^, Br^−^, or I^−^), forming a three-dimensional lattice of corner-sharing [BX_6_]^4−^ octahedra with A-site cations situated in the lattice voids.^157^ Variants of this structure can be engineered to form lower-dimensional perovskites, including 2D Ruddlesden Popper phases, 1D chains, and even 0D molecular clusters, by introducing bulky organic cations or altering stoichiometry. These dimensional modifications influence optical absorption, exciton binding energy, and environmental stability. The structural flexibility of MHPs, along with their ability to accommodate compositional variation, is key to their wide-ranging optoelectronic properties. As shown in Fig. 1, the power conversion efficiency of metal halide perovskite solar cells has experienced a remarkable increase from around 3% in 2009 to over 25% by 2025, highlighting their rapid development and potential as next-generation photovoltaic materials.

Their rise has been both rapid and extraordinary: within just over a decade, MHPs have evolved from lab-scale curiosities to materials capable of outperforming established semiconductors in a variety of optoelectronic applications.^158,159^ Their success can be attributed to a unique convergence of features: direct bandgaps suitable for visible light absorption, long carrier diffusion lengths, strong photoluminescence, defect tolerance, and low-cost solution processability. What makes MHPs particularly revolutionary is not just their intrinsic performance, but the ease with which their properties can be tailored *via* compositional engineering and dimensional modulation. Their application space is expanding rapidly, from high-efficiency solar cells to light-emitting diodes (LEDs), photodetectors, X-ray scintillators, and electrochemical energy storage devices. Meanwhile, challenges related to lead toxicity, long-term instability, and scale-up have spurred a surge in efforts toward lead-free alternatives, encapsulation technologies, and robust hybrid systems.^160^

**Fig. 2:**
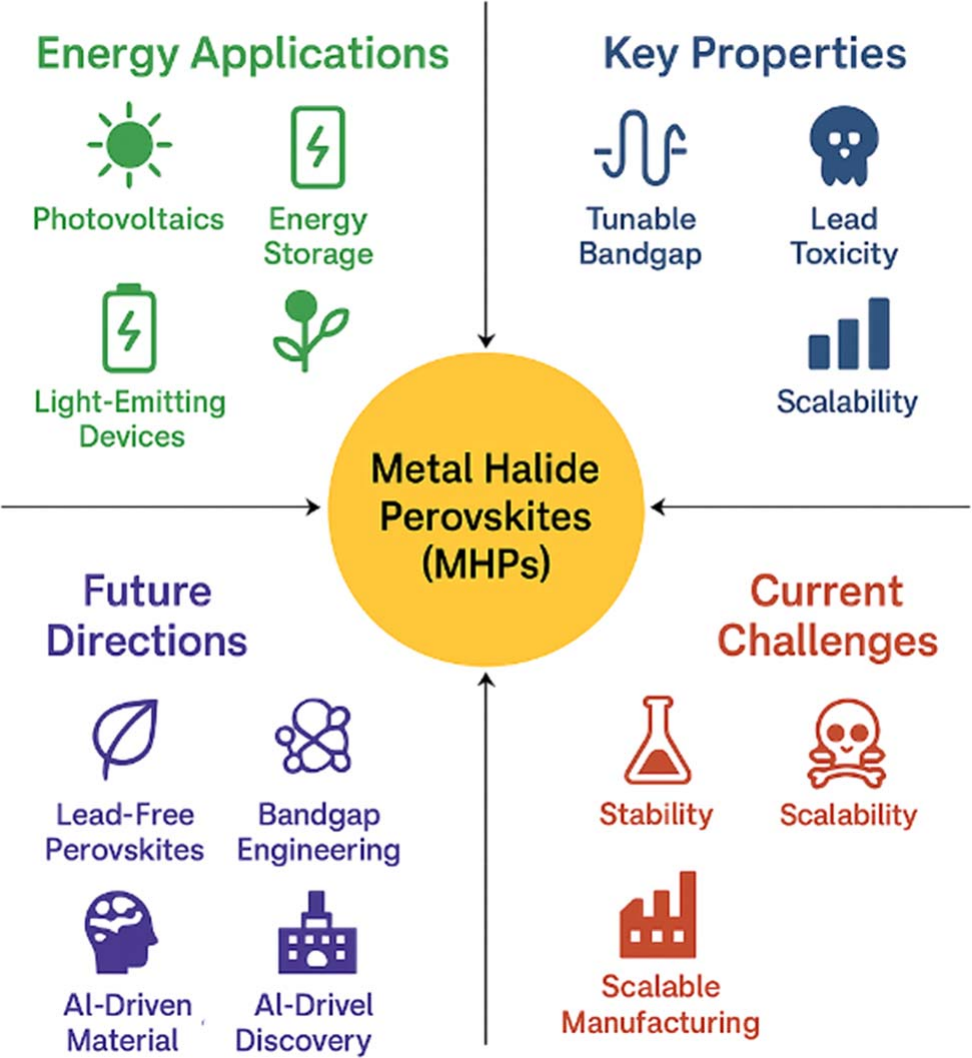
Schematic overview of metal halide perovskites: applications, properties, challenges, and future directions.

Global research activity, as seen in a surge of publications and patents, demonstrates the intense focus on understanding and extending the capabilities of MHPs across disciplines.^161,162^ Concurrently, major progress has been achieved in refining fabrication methods ranging from spin-coating and antisolvent treatments to vapor-phase deposition and scalable printing as well as in applying characterization techniques like X-ray diffraction, time-resolved photoluminescence, and transient absorption spectroscopy to study charge transport and degradation pathways. These developments are also reflected in the evolution of theoretical modelling approaches to better capture the behaviour of ion migration, exciton dynamics, and defect physics in MHPs.^163,164^ The scope of MHP research now spans device-level optimization, interfacial engineering, thermal management, and life cycle analysis all of which are critical for real-world implementation. Considering these considerations, this review provides an in-depth analysis of the recent progress in the synthesis and characterization of MHPs, focusing on their energy-related applications. Fig. 2 provides a schematic overview of metal halide perovskites, summarizing their key applications, intrinsic properties, existing challenges, and emerging future directions in energy-related technologies. We discuss various synthesis methods, ranging from solution-based techniques to vapor-phase deposition, and examine advanced characterization tools that provide insights into their structural, optical, and electronic properties. Furthermore, we highlight the key challenges that must be addressed to enhance the stability, scalability, and environmental sustainability of these materials. Finally, we explore future perspectives for expanding the applicability of MHPs beyond photovoltaics, including their potential use in thermoelectric devices, energy storage systems, and optoelectronic sensors.

## 2. Advances in synthesis and characterization of MHPs

### 2.1. Synthesis strategies

The synthesis of metal halide perovskites plays a pivotal role in determining their structural integrity, optoelectronic properties, and long-term stability. Various fabrication techniques have been developed and refined to optimize film quality, enable large-scale production, and address environmental and health concerns. This section provides an in-depth discussion of the most widely used synthesis strategies, along with a summary of their respective advantages and limitations.

#### 2.1.1 Solution processing

Solution-based methods, particularly spin-coating, dip-coating, and spray deposition, have become the most prevalent routes for perovskite film fabrication due to their low material consumption, ease of implementation, and compatibility with ambient or low-temperature processing environments.^165,166^ Among these, spin-coating remains the standard for lab-scale synthesis, allowing for rapid film deposition and enabling precise control over precursor composition and solvent dynamics. One of the key strengths of solution processing is its accessibility requiring relatively simple equipment and offering a cost-effective path to explore new formulations and additives. Furthermore, recent innovations such as antisolvent dripping, solvent engineering, and additive-assisted crystallization have significantly enhanced film uniformity, reduced defect density, and promoted larger grain sizes, all of which contribute to better device efficiency and operational stability.

However, despite these benefits, solution processing suffers from notable drawbacks. Film formation can be highly sensitive to ambient humidity and temperature, leading to batch-to-batch variation. The rapid crystallization involved in spin-coating can result in pinholes, incomplete coverage, and rough surface morphologies, particularly over large areas. Additionally, solution-derived films often contain grain boundaries and interface irregularities that can act as non-radiative recombination centers, thereby reduce power conversion efficiency and accelerate degradation under operational conditions.^167,168^ These issues make solution processing less suitable for industrial-scale production without further refinement in process control and environmental isolation. Hence, while solution-based methods offer an excellent platform for innovation and proof-of-concept devices, transitioning them to commercial-scale applications requires overcoming significant reproducibility and uniformity challenges.

#### 2.1.2 Vapor deposition techniques

Vapor-phase deposition techniques, such as thermal evaporation, chemical vapor deposition (CVD), and hybrid vapor-solution routes, offer an alternative approach to achieving highly uniform and defect-minimized perovskite films.^169,170^ These methods provide excellent control over film thickness, stoichiometry, and crystallinity, which is crucial for tandem solar cell integration and multilayer optoelectronic devices. CVD allows for conformal coating on complex geometries and is well-suited to high-throughput fabrication with atomic-scale precision. Moreover, the absence of solvent residues in vapor-deposited films results in purer interfaces, lower trap densities, and improved thermal and environmental stability compared to solution-processed counterparts.

Despite these advantages, vapor-based techniques are constrained by their inherent complexity. They typically require high-vacuum systems, multiple-source evaporation control, and tightly regulated temperature and pressure conditions, all of which contribute to increased capital and operational costs. The equipment-intensive nature of vapor deposition also limits its accessibility for exploratory research and small-scale prototyping. Additionally, scalability can be hindered by the slow deposition rates and the need for ultra-clean processing environments to maintain film integrity. While vapor techniques are promising for industrial-scale device manufacturing especially in applications demanding precision, such as tandem solar modules, they remain less cost-effective and adaptable than solution-based approaches for early-stage development and flexible electronics.^171,172^

#### 2.1.3 Green and lead-free approaches

In response to growing environmental and regulatory concerns, particularly regarding the toxicity of lead-containing perovskites, significant efforts have been directed toward the development of green and lead-free synthesis routes.^173,174^ Green processing strategies emphasize solvent systems that are less hazardous or recyclable, aim to reduce waste, and often incorporate ambient or low-energy processing conditions. For example, replacing highly toxic solvents like dimethylformamide (DMF) with greener alternatives such as γ-butyrolactone (GBL), dimethyl sulfoxide (DMSO), or water-based formulations represents a step toward safer and more sustainable fabrication. Additionally, innovations in solvent-free deposition methods and *in situ* crystallization during film growth have shown potential in reducing environmental impact.^175^

**Fig. 3:**
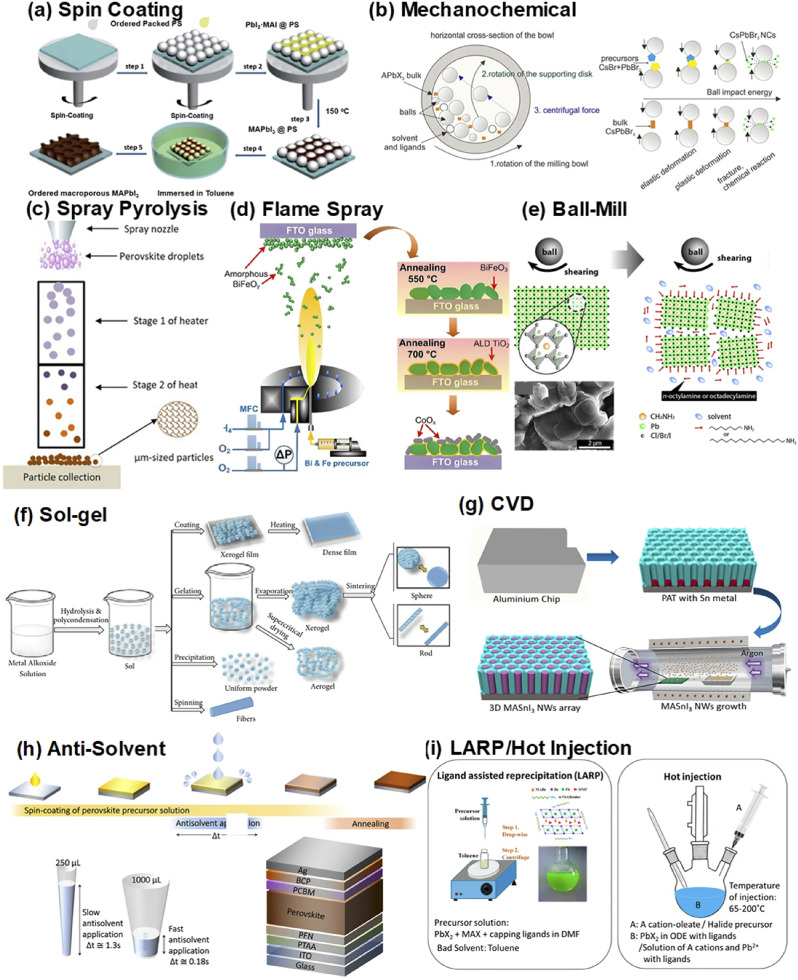
Different techniques for synthesizing perovskite and nanocomposite structures: (a) spin coating, (b) mechanochemical synthesis, (c) spray pyrolysis, (d) flame spray pyrolysis, (e) ball milling, (f) sol–gel method, (g) chemical vapor deposition (CVD), (h) antisolvent approach, and (i) ligand-assisted reprecipitation (LARP) with hot injection.^178^

Parallel to green processing, lead-free perovskite alternatives such as tin-based (*e.g.*, MASnI_3_) and double perovskite structures (*e.g.*, Cs_2_AgBiBr_6_) have been widely investigated. These materials offer a non-toxic substitute for Pb^2+^ and align more closely with environmental safety guidelines.^176^ However, the performance of lead-free perovskites has lagged behind due to their inherent instability tin, for instance, is prone to oxidation from Sn^2+^ to Sn^4+^, which introduces deep-level trap states and degrades optoelectronic properties. Double perovskites, while more stable, often exhibit indirect bandgaps and lower carrier mobility, limiting their effectiveness in high-efficiency photovoltaic applications. Moreover, the phase purity and crystallinity of these materials remain a synthesis challenge. Despite these limitations, green and lead-free approaches are essential for ensuring the long-term commercial viability and public acceptance of perovskite technologies, and ongoing work in doping, surface passivation, and hybrid organic–inorganic systems continues to narrow the performance gap.^177^ As shown in Fig. 3, a wide range of synthesis techniques including spin coating, mechanochemical synthesis, and spray pyrolysis are utilized to fabricate perovskite and nanocomposite materials with tailored properties.^178^

### 2.2. Characterization techniques

The comprehensive characterization of MHPs is crucial to understanding their structural, optical, and stability-related properties, which are key to optimizing performance and ensuring long-term durability.

#### 2.2.1 Structural analysis

Techniques such as X-ray diffraction (XRD) and Raman spectroscopy are fundamental in assessing the crystallinity, phase purity, and stability of MHPs. XRD provides insights into lattice parameters and phase composition, while Raman spectroscopy helps in detecting structural distortions and identifying characteristic vibrational modes, crucial for understanding phase transitions and defect states.^179–184^

#### 2.2.2 Optoelectronic properties

Evaluating the optoelectronic performance of MHPs involves techniques like UV-vis spectroscopy, photoluminescence (PL), and time-resolved PL. UV-vis spectroscopy is used to determine optical bandgaps and absorption profiles, while PL and time-resolved PL offer insights into charge carrier dynamics, recombination processes, and material quality. These analyses help in correlating the synthesis conditions with optoelectronic performance.^185–187^

#### 2.2.3 Thermal and environmental stability

Stability assessments are critical due to the degradation sensitivity of MHPs. Thermogravimetric analysis (TGA) helps determine thermal decomposition behaviours, while humidity-dependent stability tests assess material performance under varying environmental conditions. Understanding these degradation pathways enables the design of more robust materials and encapsulation strategies.

#### 2.2.4 Advanced imaging

**Fig. 4:**
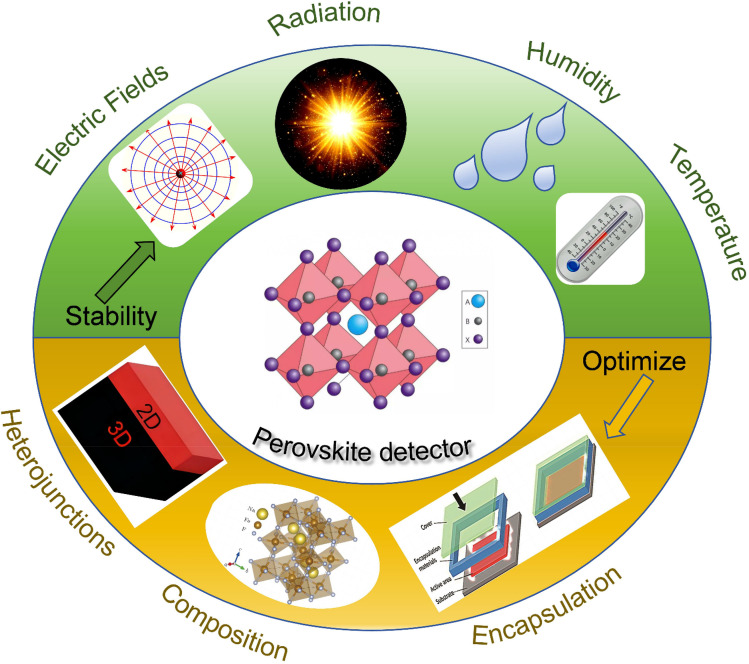
Common synthesis routes and characterization techniques for metal halide perovskites.^188^

Scanning electron microscopy (SEM) and transmission electron microscopy (TEM) are pivotal in investigating morphological features, grain structures, and interfacial properties. SEM provides detailed surface morphology, identifying defects and pinholes, while TEM offers atomic-level insights into crystal structures and interfacial characteristics. These imaging techniques are essential for optimizing film quality and enhancing device performance. Fig. 4 illustrates the various synthesis techniques and characterization methods used for Metal Halide Perovskite.^188^ It highlights the main approaches, such as solution processing, vapor deposition, and lead-free alternatives, alongside essential characterization techniques, including XRD, Raman spectroscopy, and UV-vis spectroscopy. The arrows and lines represent the flow from synthesis to characterization, demonstrating how each method contributes to optimizing MHPs for various applications.

## 3. Challenges and future directions

### 3.1. Stability concerns

#### 3.1.1 Moisture sensitivity

MHPs are highly sensitive to moisture, which significantly impacts their stability and performance. In humid environments, water molecules can infiltrate the perovskite lattice, causing the material to degrade, which in turn reduces the efficiency of devices such as solar cells. This moisture-induced degradation is one of the primary obstacles for large-scale commercialization of MHP-based technologies. To mitigate this, several strategies have been explored, including encapsulation techniques such as coating the perovskite layer with moisture-resistant materials like polymers or glass. Additionally, compositional engineering, modifying the chemical makeup of the perovskite itself, can enhance water resistance. For instance, incorporating fluoride or phosphates in the precursor solution has shown potential in improving the material's resistance to moisture.^189–191^ However, achieving long-term stability without compromising performance remains a significant challenge.

#### 3.1.2 Thermal decomposition

Thermal instability is another major issue for MHPs, particularly for devices exposed to high operating temperatures. Organic cations used in hybrid perovskites (like methylammonium or formamidine) are prone to decomposition under heat, leading to a reduction in the perovskite's crystallinity and electrical performance. This thermal degradation restricts the operational temperature range, which is critical for applications in environments where temperature fluctuations are common. Research efforts are focusing on all-inorganic perovskites such as caesium lead halide (CsPbX_3_), which show significantly better thermal stability due to the absence of organic cations. While these materials are more stable, they still face challenges such as lower efficiency and structural defects, which limit their commercial viability. Further advancements in material design, such as exploring different metal halides and doping strategies, are required to achieve both high stability and performance.^192–194^

#### 3.1.3 UV sensitivity

In addition to moisture and thermal degradation, UV sensitivity poses another challenge for perovskites, particularly in solar cell applications. UV radiation can lead to the breakdown of the perovskite structure, causing loss in efficiency over time. Efforts are underway to explore UV-resistant coatings or to modify the perovskite composition to enhance its UV stability. Additionally, the use of protective layers and encapsulants that shield the device from UV exposure is also under investigation.^195^

#### 3.1.4 Hysteresis and ion migration

Another important factor affecting the long-term performance of MHP-based devices is ion migration within the perovskite material. This migration can lead to a phenomenon known as hysteresis, where the device's current–voltage characteristics change depending on the scan direction, causing instability in performance. Ion migration occurs due to the high ionic mobility in perovskites, which can alter the distribution of charges and electric fields inside the material. Research has suggested that controlling the ion migration through material engineering, such as using dopants or optimizing the grain boundary structure, could help mitigate this issue. Reducing hysteresis is crucial for the reliable operation of perovskite-based devices, especially for energy storage applications, where consistency over time is paramount.^196,197^

**Fig. 5:**
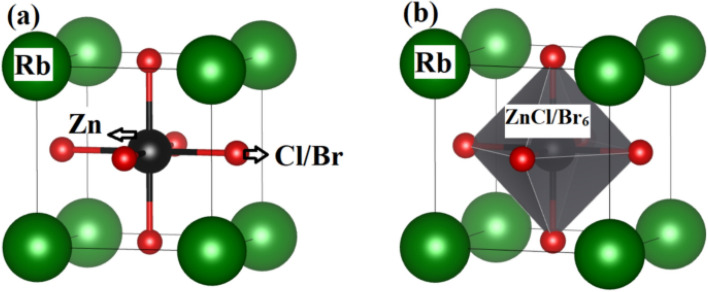
(a) Crystal structure (b) octahedra of ZnCl_6_/Br_6_ of RbZnX_3_ (X = Cl, Br) perovskites.^198^

Naimat *et al.*^198^ conducted a theoretical investigation into the structural and optoelectronic properties of RbZnX_3_ (X = Cl, Br) halide perovskites, emphasizing their potential for sustainable energy applications. The study confirmed that these materials crystallize in a face-centered cubic phase (*Fm*3̄*m*) and demonstrated their geometrical, thermodynamic, mechanical, dynamic, and thermal stability. Using the Birch–Murnaghan equation of state, they achieved energy minimization and calculated equilibrium lattice parameters. The results, illustrated in Fig. 5, indicated that the lattice constants increased with the ionic radius, resulting in a larger unit cell volume for RbZnBr_3_ compared to RbZnCl_3_. Furthermore, electronic band structure calculations revealed that both compounds are semiconductors with band gaps of 1.34 eV and 0.12 eV, respectively. The materials also exhibited strong absorption in the visible and ultraviolet spectra, with absorption coefficients reaching 10^4^ cm^−1^, indicating their suitability for photovoltaic and optoelectronic applications.^198^

**Fig. 6:**
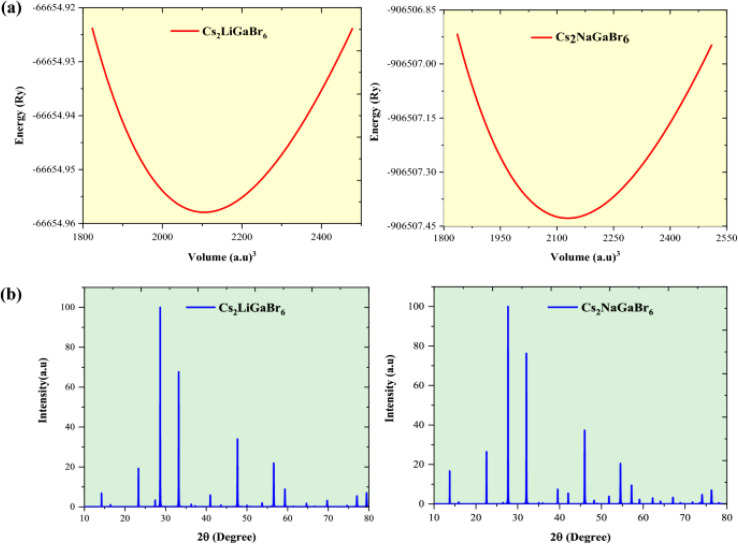
(a) Graphical depiction of the optimized volume–energy relationship, and (b) DFT-simulated XRD patterns of Cs_2_MGaBr_6_ (M = Li, Na) double halide perovskites.^199^

Mudasir *et al.*^199^ explored the lead-free halide double perovskites Cs_2_MGaBr_6_ (M = Li, Na) for sustainable energy applications. Utilizing density functional theory (DFT) and post-DFT techniques, the study assessed the materials' structural stability, transport properties, and electron phonon interactions. Structural optimization *via* the GGA-PBE potential confirmed the stability of the pristine structures. The electronic properties, calibrated with spin orbit coupling (SOC) effects, revealed band gaps of 1.82 eV for Cs_2_LiGaBr_6_ and 1.78 eV for Cs_2_NaGaBr_6_ both within the visible spectrum. Thermal transport characteristics, including phonon behavior and electron phonon coupling, were systematically analyzed, revealing a strong coupling strength supported by the Fröhlich coupling constant and Feynman polaron radius. The thermoelectric figure of merit (*zT*) values of 1.08 and 1.04 for Cs_2_LiGaBr_6_ and Cs_2_NaGaBr_6_, respectively, highlight their potential in renewable energy applications. Additionally, the materials demonstrated high optical absorption coefficients in the visible and infrared spectra, as shown in Fig. 6, supporting their suitability for optoelectronic and solar cell technologies.^199^

**Fig. 7:**
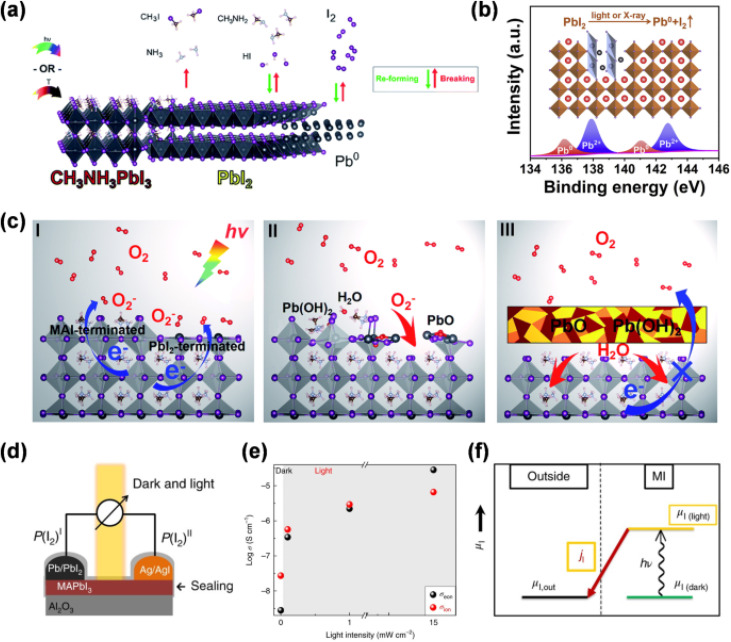
(a) The photodecomposition and thermal degradation of MAPbI_3_ lead to irreversible decomposition into organic volatile gas species (CH_3_I + NH_3_), reversible decomposition (CH_3_NH_2_ + HI), and the reversible formation of I_2_ and non-volatile Pb_0_ when exposed to light or mild heat conditions. (b) Under light or X-ray exposure, MAPbI_3_ decomposes, forming Pb_0_ and I_2_, which are associated with iodine vacancies. (c) A schematic of the photo-oxidative degradation process of MAPbI_3_. (d) An open-circuit voltage battery cell where MAPbI_3_ serves as the solid electrolyte. (e) A DC galvanostatic polarization experiment conducted at 40 °C in an Ar atmosphere to distinguish between ionic (*σ*_ion_) and electronic (*σ*_eon_) contributions. (f) The change in the chemical potential diagram of iodine under light exposure, deviating from its equilibrium value MI refers to metal iodide and “outside” refers to zero iodine concentration.^200^

Seonhong *et al.*^200^ investigated the intrinsic nature of lead halide perovskites, focusing on their stability under various conditions and their photoelectrochemical properties. The study emphasizes the challenges related to the instability of halide perovskites, especially under photoirradiation and electric fields. Photoexcitation of halide perovskites leads to ion migration, such as the formation of iodine vacancies, which result in structural and functional disruptions. These disruptions contribute significantly to the degradation of the material, with Pb_0_ formation through photoreduction being a primary decomposition product. This process is depicted in Fig. 7, where defect centers, represented by Pb_0_, inhibit charge transfer and enhance non-radiative recombination, diminishing the performance of perovskite solar cells (PSCs). Additionally, exposure to oxygen and moisture accelerates degradation through the formation of superoxide ions, which react with the generated carriers and further break down the material. The study also discusses the impact of light exposure on ion migration, highlighting a decrease in the migration energy barrier, which weakens the material and alters its electronic properties. The migration of ion vacancies to the interface between the hole transport layer and the perovskite layer forms a Debye layer that hinders charge extraction, further reducing device efficiency.

### 3.2. Toxicity and environmental impact

Metal halide perovskites have emerged as promising candidates for next-generation energy technologies, especially in photovoltaics, due to their excellent optoelectronic properties and low-cost fabrication. However, the presence of toxic elements, particularly lead (Pb), raises significant environmental and health concerns, threatening the long-term sustainability of these materials. Addressing these issues is crucial for enabling safe and responsible commercialization.

#### 3.2.1 Lead-free alternatives

Lead-based perovskites, such as MAPbI_3_, have achieved impressive efficiencies but raise serious environmental and health concerns due to lead toxicity. To mitigate this, research has focused on developing lead-free alternatives like tin (Sn), germanium (Ge), bismuth (Bi), and antimony (Sb)-based perovskites. Tin-based compounds, such as CH_3_NH_3_SnI_3_, show potential but suffer from rapid oxidation, leading to instability. Strategies like adding reducing agents, using surface passivation, and applying encapsulation techniques are being explored to enhance stability. Germanium-based perovskites also face oxidation challenges but offer better environmental compatibility. Meanwhile, Bi and Sb-based materials, including Cs_2_AgBiBr_6_, provide greater stability and lower toxicity but still require improvements in charge transport and efficiency. Future research should focus on compositional engineering, hybrid material designs, and the development of novel, earth-abundant, non-toxic materials to balance performance and environmental safety.

**Fig. 8:**
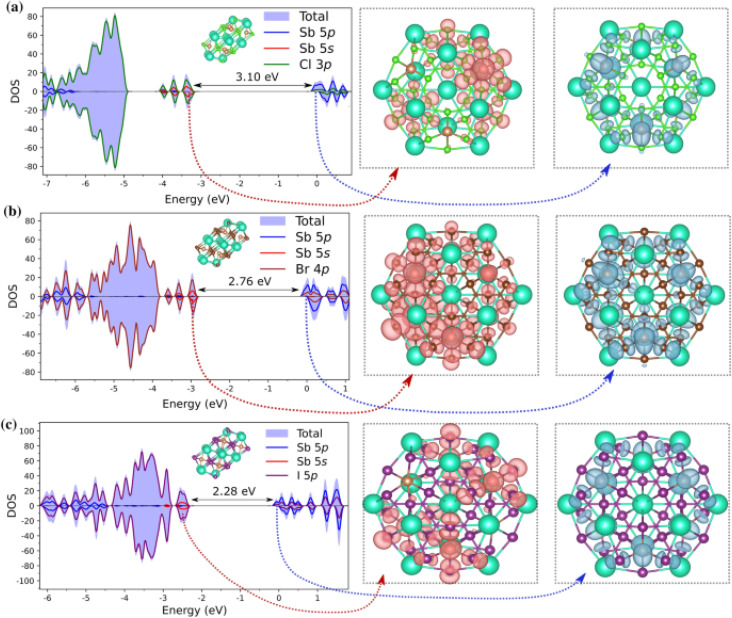
Partial density of states (PDOS) and frontier molecular orbitals for Cs_13_Sb_6_X_30_ clusters, where X = Cl, Br, and I. Panels (a), (b), and (c) correspond to the halides Cl, Br, and I, respectively. The total and atomic orbital contributions (Sb 5p, Sb 5s, and halogen p orbitals) to the density of states are shown on the left. The highest occupied molecular orbital (HOMO) and lowest unoccupied molecular orbital (LUMO) distributions are visualized on the right for each composition, with the energy band gaps indicated between the HOMO and LUMO levels.^201^

In their study, Gouvêa *et al.*^201^ conducted a comprehensive density functional theory (DFT) analysis of lead-free cesium antimony halide perovskites (Cs_3_Sb_2_X_9_, where X = Cl, Br, I), focusing on halide alloying, structural modifications, and surface characteristics. They evaluated the enthalpy of formation and miscibility gap temperatures to determine the feasibility of synthesizing various solid solutions of 
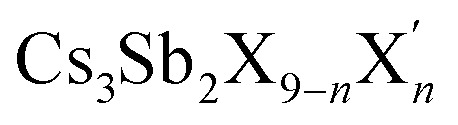
. Their findings indicated that CsX-terminated low-index (1000) and (0001) surfaces exhibit unique electronic properties, highlighting the significance of surface control during material preparation for optimizing photovoltaic and photocatalytic applications. Cluster simulations of Cs_3_Sb_2_X_9_ nanocrystals revealed that geometric factors may contribute to the high photoluminescence observed in previous experimental studies on Cs_3_Sb_2_Br_9_ nanocrystals. Notably, the partial density of states (PDOS) presented in Fig. 8 illustrates the electronic structure of Cs_13_Sb_6_X_30_ clusters, showing how spatial confinement leads to larger band gaps compared to bulk materials. The study also suggested that combining Cs_3_Sb_2_Br_9_ with Cs_3_Sb_2_I_9_ is promising for photovoltaic applications, while pairing Cs_3_Sb_2_Br_9_ with Cs_3_Sb_2_Cl_9_ could enhance photoluminescence, based on their band alignment and electronic structures. These insights advance the understanding of lead-free Cs_3_Sb_2_X_9_ perovskites and provide practical guidance for designing cesium antimony halide perovskites with tailored optical and electronic properties, supporting the development of sustainable energy solutions for optoelectronic devices.

#### 3.2.2 Recycling and waste management

The environmental risks associated with lead leakage from perovskite solar cells (PSCs) emphasize the need for efficient recycling and waste management. Current strategies focus on recovering valuable materials like lead through solvent extraction, thermal treatment, and mechanical separation. Eco-friendly recycling approaches aim to establish closed-loop systems where recovered materials are reused in new devices, reducing environmental impact and promoting sustainability. Designing devices with easily separable layers and fewer hazardous components can enhance recyclability. Additionally, using advanced encapsulation materials can minimize leakage during disposal. Regulatory frameworks and lifecycle assessments are also essential to ensure safe handling and processing, while promoting design-for-recycling principles can lead to more sustainable perovskite technologies.

**Fig. 9:**
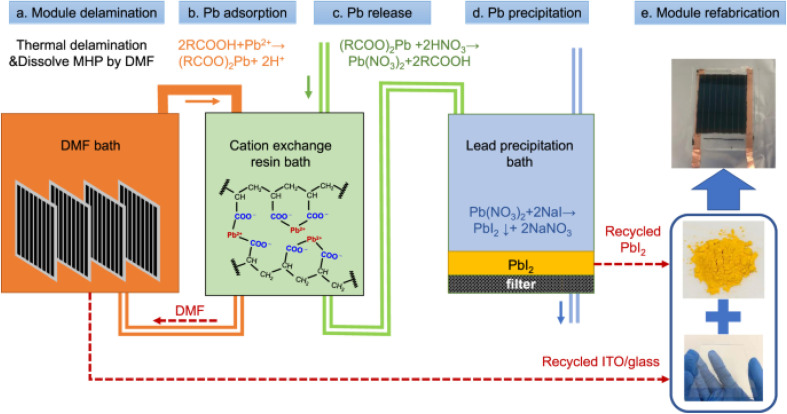
(a) Encapsulated perovskite solar modules were delaminated and MHP was dissolved by DMF. (b) Lead ions in DMF were removed by carboxylic acid cation-exchange resin. (c) The adsorbed lead ions on resin were released to aqueous solution by resin-regeneration process *via* HNO_3_. (d) Precipitation of PbI_2_ by pouring NaI into Pb(NO_3_)_2_ containing solution. (e) Module refabrication based on recycled materials.^202^

Chen *et al.*^202^ developed a cost-effective waste management strategy for perovskite solar modules, focusing on the recycling of toxic lead and valuable transparent conductors to mitigate environmental impact and generate economic benefits. The process involves separating lead from decommissioned modules using a weakly acidic cation-exchange resin, achieving a high recycling efficiency of 99.2%. The lead is first extracted as soluble Pb(NO_3_)_2_ and then precipitated as PbI_2_ for reuse. Additionally, thermal delamination is employed to disassemble the encapsulated modules, preserving the transparent conductors and cover glasses. The refabricated devices using recycled materials exhibit comparable performance to those made with fresh raw materials. This process, illustrated in the recycling roadmap (Fig. 9), also details the dissolution of lead from the perovskite layer using an organic solvent (dimethylformamide, DMF), followed by lead adsorption, release, and precipitation as PbI_2_ for reuse. The study emphasizes the environmental and economic advantages of recycling lead and transparent conductors from perovskite solar modules, making this approach a promising solution for the sustainable disposal of perovskite-based devices.

#### 3.2.3 Regulatory framework and lifecycle assessments

Establishing strong regulatory frameworks is essential to ensure the safe production, use, and disposal of perovskite materials, particularly those containing toxic elements like lead. Regulations should focus on safe handling, transportation, and waste management practices while encouraging manufacturers to adopt sustainable production methods. Lifecycle assessments (LCA) are critical for evaluating the environmental impact of perovskite solar cells from material extraction to end-of-life disposal. These assessments help identify areas where environmental risks can be minimized and support the development of eco-friendly materials and processes. Implementing LCA early in the design phase can drive innovations that reduce environmental footprints, ensuring the long-term sustainability of perovskite technologies.

#### 3.2.4 Emerging approaches to minimize environmental impact

Innovative approaches are being explored to reduce the environmental impact of perovskite solar cells. One strategy is the development of encapsulation technologies that prevent lead leakage, even if the device is damaged or degrades over time. Another approach involves designing perovskites with less hazardous components, including the use of biodegradable or recyclable materials for supporting layers. Research into solvent-free and low-energy synthesis methods also aims to reduce the carbon footprint of production. Additionally, advancements in device architecture, such as multi-layer structures that enhance stability and facilitate material recovery, are being investigated. These strategies, combined with policies promoting green chemistry and sustainable design, can significantly lower the environmental impact of perovskite technologies.

### 3.3. Scalability and commercialization

The scalability and commercialization of perovskite-based technologies face several key challenges that must be addressed for widespread industrial adoption. One critical area is the large-scale fabrication of perovskite devices, where methods such as roll-to-roll printing and scalable deposition techniques need further refinement. These techniques, which offer the potential for cost-effective mass production, must ensure uniformity and high performance across large surfaces. Optimization of these processes is essential to enable cost reductions and enhance throughput for commercial-scale manufacturing. Additionally, achieving long-term stability remains a major hurdle for perovskite-based devices. The perovskite materials themselves can degrade over time due to moisture, oxygen exposure, and thermal cycling, leading to a reduction in efficiency. To ensure the commercial viability of perovskite solar cells and other devices, robust device encapsulation methods must be developed to protect the perovskite layer. Moreover, interface engineering plays a crucial role in enhancing the stability and performance of perovskite devices. This includes improving the charge transport properties and minimizing defects at the interfaces between the perovskite layer and other components. These advancements are necessary to guarantee that perovskite-based devices perform consistently over their intended lifespan, thus paving the way for their successful commercialization.

**Fig. 10:**
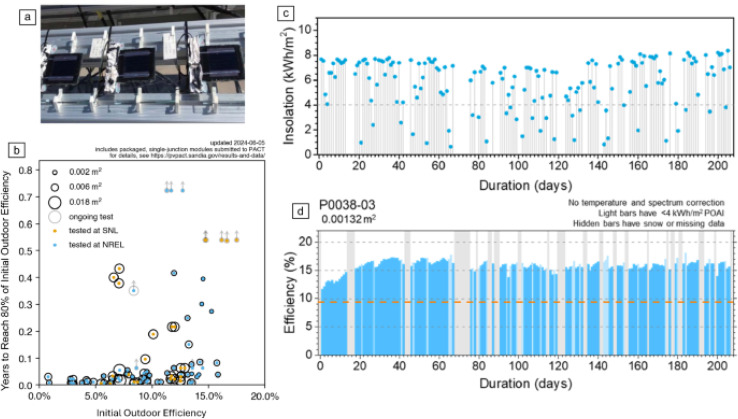
Outdoor field stability test results. Pictures of modules installed outdoor (a). Outdoor stability test results at the perovskite PV Accelerator for Commercializing Technologies (PACT) (b). SNL, Sandia National Laboratories; NREL, National Renewable Energy Laboratory. Light intensity (c) and resulting device performance of one of the modules at PACT (d).^203^

Benjia *et al.*^203^ investigate the commercialization of perovskite-based photovoltaic technology, which is progressing rapidly towards becoming a viable product. With efficiencies exceeding 26%, multiyear outdoor durability tests, and large-scale production plans for full-area panels up to 2 m^2^, the technology shows great potential. However, for perovskites to compete in the photovoltaic market and contribute to global energy solutions, the technology must achieve high efficiency, durability, and scalability. The study highlights the challenges in achieving long-term stability and durability, particularly when comparing perovskite solar modules to conventional photovoltaic technologies. One key challenge is the limited operational lifespan of perovskite modules, which still fall short of the 25 to 30-year reliability standards typical of commercial photovoltaic modules like those produced by First Solar. Fig. 10 illustrates the outdoor field stability test results, showing the performance of perovskite modules under real-world conditions. Despite impressive efficiency gains, the ability to maintain performance over extended periods remains a significant hurdle. To be commercially viable, perovskite modules must endure environmental stresses such as UV light, heat, and water exposure.^203^ While packaging advancements are being made to prevent water and oxygen ingress, improvements in the materials and device cohesion are needed to ensure long-term durability, particularly in large-area devices.

### 3.4. Advanced characterization for performance optimization

The rapid progress in metal halide perovskite materials for energy applications, particularly in solar cells, has made advanced characterization techniques crucial for optimizing device performance. To achieve the full potential of these materials, a deeper understanding of their behavior under real-world conditions is essential. Recent developments in characterization techniques have provided valuable insights into material properties, degradation pathways, and charge transport mechanisms, ultimately improving the efficiency, stability, and scalability of perovskite-based energy devices.

#### 3.4.1 Machine learning for material discovery

As the field of perovskite solar cells continues to evolve, machine learning has become an indispensable tool for accelerating the discovery of new perovskite compositions. Machine learning algorithms enable high-throughput screening of a vast range of potential material combinations, significantly reducing the time and resources needed for experimental trials. By analyzing large datasets, machine learning can identify patterns that link material properties to performance outcomes, guiding researchers to new material formulations with enhanced stability and efficiency. For instance, machine learning can predict the stability of perovskite compositions under various environmental conditions, which is a critical factor in commercializing perovskite-based devices. By optimizing the discovery process, machine learning opens the door to designing more efficient and durable perovskite materials for energy applications.

Moreover, the integration of high-throughput screening methods has enabled the rapid evaluation of a wide variety of halide combinations, which is essential for discovering the optimal balance of properties such as bandgap, carrier mobility, and defect tolerance. These advancements will play a pivotal role in overcoming the stability issues that have limited the commercial success of metal halide perovskites. Gang Li *et al.*^204^ explore the application of machine learning for the rapid discovery of narrow-bandgap inorganic halide perovskite materials, a key factor in optimizing solar cell efficiency. While density functional theory (DFT) can be used to calculate the bandgap of materials, it is often slow and constrained by complex electronic correlations and lattice dynamics, leading to discrepancies between theoretical and experimental results. To overcome these limitations, the study utilizes machine learning techniques, specifically the XGBoost classifier, to predict the bandgap of inorganic halide perovskites. The authors compiled a dataset of 447 perovskites and used the Matminer Python package to generate material descriptors. The model achieved an impressive 95% accuracy in predicting narrow-bandgap materials.

**Fig. 11:**
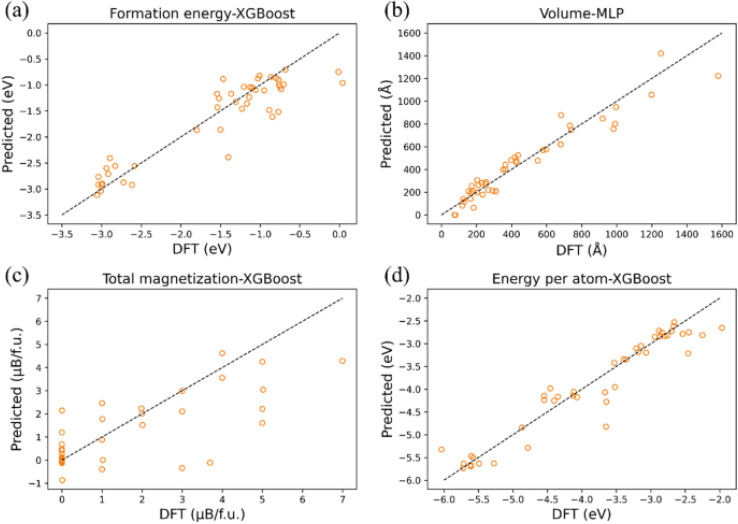
The best algorithm fitting graph for the four properties on the test set. (a) XGBoost for formation energy. (b) MLP for volume. (c) XGBoost for total magnetization. (d) XGBoost for energy per atom.^204^

In addition, the study employed Shapley analysis to identify the factors influencing the bandgap. The analysis revealed that the electronegativity range is the most significant factor: as the range increases, so does the likelihood of obtaining a narrow-bandgap perovskite. These findings highlight the potential of machine learning in accurately predicting perovskite properties with speed and precision, which could significantly accelerate the discovery of new materials. As shown in Fig. 11, the study demonstrates that XGBoost had smaller errors in predicting formation energy, total magnetization, and energy per atom. In contrast, the MLP algorithm exhibited smaller errors in predicting volume. Overall, by predicting multiple material properties, the machine learning model provides a more comprehensive understanding of material characteristics, thereby enhancing its practical utility.^204^

#### 3.4.2 *In situ* and *operando* studies

*In situ* and *operando* characterization techniques have become indispensable tools in the study of metal halide perovskites, particularly in understanding their complex degradation mechanisms under real-world conditions. These materials, while promising for optoelectronic applications like solar cells and LEDs, often suffer from instability when exposed to light, heat, moisture, and electric fields. Traditional *ex situ* methods, which analyse materials before and after exposure, fail to capture the transient phenomena that occur during actual operation.^205^ In contrast, *in situ* techniques allow researchers to probe changes in material structure or properties under controlled environmental conditions such as humidity or illumination while *operando* techniques go a step further by examining these changes in real-time as the device functions, such as during power generation in a working solar cell.^206^

The insights provided by *in situ* and *operando* methods are crucial in uncovering dynamic processes like ion migration, phase segregation, and interface reactions, which significantly affect device performance and longevity. For instance, *operando* photoluminescence (PL) and X-ray diffraction have revealed reversible formation of defect states and transient phase changes under continuous illumination. Techniques such as environmental transmission electron microscopy and time-resolved spectroscopy enable visualization of grain boundary evolution, PbI_2_ formation, and perovskite decomposition with high spatial and temporal resolution. Such observations are vital to distinguishing between reversible effects those that can be mitigated with design improvements and irreversible ones that fundamentally limit device stability.^207^

However, these techniques are not without challenges. Maintaining realistic operational conditions such as illumination, electrical bias, or atmospheric composition within the confines of advanced instrumentation can be technically complex. There is often a trade-off between spatial resolution and device relevance, especially in vacuum-based tools like TEM, where beam-induced damage and altered material behavior under low-pressure conditions can skew results. Despite these limitations, the complementary nature of *in situ* and *operando* approaches helps build a holistic understanding of perovskite behavior, offering both mechanistic insights and practical guidance for material and device engineering.

The knowledge gained from these studies has already influenced perovskite device design strategies. For example, understanding moisture-induced degradation has driven the development of better encapsulation methods, while insights into ion migration have led to the use of mixed-cation or mixed-halide compositions that suppress instabilities. Interface engineering, including passivation layers and buffer materials, has also benefited from *operando* studies showing how defects form and evolve at contacts under stress. Furthermore, the field is moving toward integrating multiple *in situ* tools such as coupling PL with electrical measurements or XRD with the aid of machine learning for data analysis, enabling faster and more accurate identification of degradation patterns.^208^

Looking ahead, the continued advancement of *in situ* and *operando* methodologies will be critical for pushing metal halide perovskites toward commercial viability. Standardizing these techniques across research groups will improve reproducibility and foster deeper collaboration. As these methods become more sophisticated, they will not only reveal how perovskites fail but also guide the design of next-generation materials and architectures that are robust, efficient, and ready for deployment in real-world environments.

#### 3.4.3 Charge transport and defect passivation

One of the most important factors influencing the efficiency and longevity of perovskite-based energy devices is the effective transport of charge carriers within the material. Defects such as vacancies, grain boundaries, and interfaces often act as traps for charge carriers, reducing the overall efficiency of perovskite solar cells. Therefore, understanding the role of these defects and developing strategies for defect passivation is crucial for optimizing charge transport and improving device performance. Recent research has focused on molecular engineering, which involves designing and incorporating specific passivating agents that interact with defects to neutralize their harmful effects. Interface engineering is another promising approach to enhance charge transport by improving the interaction between the perovskite layer and the charge transport layers. By optimizing the interfaces, researchers can minimize charge recombination and increase the efficiency of charge extraction. Additionally, doping strategies have been employed to improve charge mobility, reduce recombination losses, and enhance the stability of perovskite films.

The understanding of defect dynamics has also led to the development of strategies that mitigate the impact of defects on device performance. These include the use of new additives that prevent defect formation and stabilize the crystal structure under stress. By integrating these strategies, researchers aim to increase the power conversion efficiency (PCE) of perovskite solar cells and extend their operational lifespan.

### 3.5. Emerging applications beyond photovoltaics

The recent developments in Metal Halide Perovskites have expanded their applications well beyond traditional photovoltaic devices. MHPs' unique properties, such as tunable optoelectronic characteristics, low thermal conductivity, and high ionic conductivity, have led to their exploration in a variety of advanced technologies, which promise to significantly enhance energy conversion, storage, and sensing capabilities. In this section, we delve into some of the most exciting emerging applications for MHPs.

#### 3.5.1 Thermoelectric and energy harvesting applications

One of the most promising directions for MHPs is their application in thermoelectric devices, which can convert waste heat into usable electricity. Thermoelectric materials are evaluated based on their Seebeck coefficient, electrical conductivity, and thermal conductivity. MHPs are particularly attractive for this purpose due to their low thermal conductivity and high Seebeck coefficients. These characteristics allow MHPs to maintain a significant temperature gradient, making them ideal for thermoelectric conversion. As a result, MHP-based thermoelectric materials could play a critical role in waste heat recovery technologies, where they can capture and convert energy from industrial processes, vehicle exhausts, or even human body heat into usable power. The potential for MHPs to enhance the efficiency of thermoelectric devices is not only a promising step toward sustainable energy but also presents a low-cost solution for energy harvesting applications, paving the way for self-powered devices in sectors such as electronics, automotive, and aerospace.

Hanof Dawas Alkhaldi *et al.*^209^ investigate the electronic, mechanical, optical, and thermoelectric properties of halide double perovskites (DPH) Na_2_AuInZ_6_ (Z = Cl, Br, I) with the potential for solar cells and renewable energy applications. The study focuses on the optoelectronic and thermoelectric properties of these materials to evaluate their suitability for energy devices. The cubic-phase DPHs are found to be structurally stable based on computed structural and elastic properties, with formation energy calculations confirming their thermodynamic stability. The ductile nature of these materials is also suggested, as indicated by Pugh's and Poisson's ratios. To accurately assess the optoelectronic characteristics, the Tran–Bhala modified Becke and Johnson potential (TB-mBJ) is used. The study reveals that these compounds exhibit direct band gaps of 2.70 eV, 1.75 eV, and 0.46 eV, making them viable for various optoelectronic applications. The optical properties, such as absorption, reflectance, and energy loss, are analysed for potential solar cell applications within the incident light energy range of 0–6 eV. The thermoelectric characteristics are evaluated by considering the effect of temperature, with power factor and figure-of-merit analysis indicating that these materials may be suitable for thermoelectric devices. Thus, this study comprehensively highlights the potential of halide perovskites in sustainable energy technologies.

**Fig. 12:**
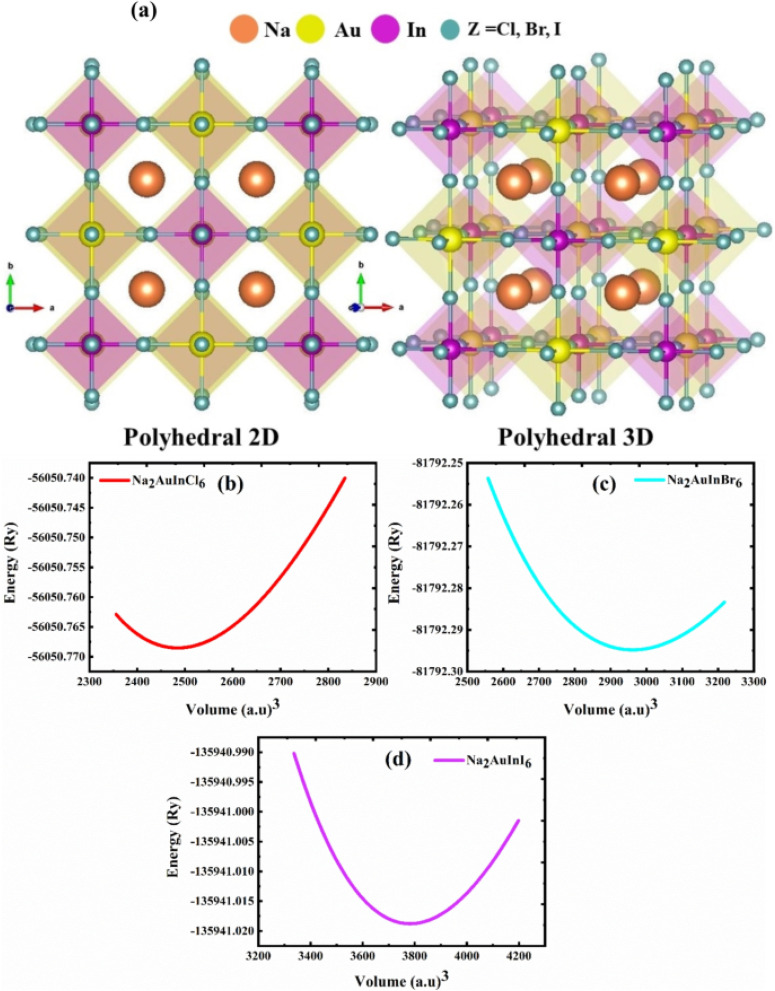
(a) Atomic and polyhedral structures of 2D and 3D cubic Na_2_AuInZ_6_ perovskites, where Z = Cl, Br, I. Sodium (Na), gold (Au), indium (In), and halogen atoms are represented by orange, yellow, violet, and cyan spheres, respectively. The polyhedral network illustrates the octahedral coordination around the metal cations. (b–d) Energy–volume optimization curves for (b) Na_2_AuInCl_6_, (c) Na_2_AuInBr_6_, and (d) Na_2_AuInI_6_.^209^

Regarding the structural properties, the Na_2_AuInZ_6_ (Z = Cl, Br, I) perovskites exhibit a cubic unit cell, as shown in Fig. 12, belonging to the space group *Fm*3̄*m* (no. 225). The lattice constants (*a*_0_) for Na_2_AuInCl_6_, Na_2_AuInBr_6_, and Na_2_AuInI_6_ are 11.37, 12.06, and 13.08 Å, respectively. The increasing lattice constant is attributed to the larger ionic radii of halogens (Cl < Br < I). Iodine-based perovskites have a higher lattice constant than chlorine- and bromine-based ones, likely due to the larger ionic radius of iodine. The bulk modulus (*B*), which measures a material's resistance to volume changes under pressure, shows an inverse correlation with the lattice constant values. As a result, Na_2_AuInCl_6_, which has the largest bulk modulus, is the most solid and structurally rigid material among the compounds studied. The stability of these materials is also supported by their tolerance factor and octahedral factor, which confirm the compounds' stability and formation potential. Danish Abdullah *et al.*^210^ investigate the structural, optoelectronic, and thermoelectric properties of lead-free fluoride perovskites A_2_GeSnF_6_ (A = K, Rb, Cs) using density functional theory. The study confirms the dynamical stability of these compounds through a stable phonon dispersion spectrum, while the enthalpy of formation and tolerance factor further verify their structural stability. The predicted direct band gaps are 3.19 eV for K_2_GeSnF_6_, 3.16 eV for Rb_2_GeSnF_6_, and 3.12 eV for Cs_2_GeSnF_6_. These results suggest that A_2_GeSnF_6_ (A = K, Rb, Cs) double perovskites are promising candidates for optoelectronic devices due to their suitable bandgaps. The calculated figure of merit values (0.94–0.97) highlights the potential of these materials for thermoelectric devices, underlining their prospective application in energy harvesting.

**Fig. 13:**
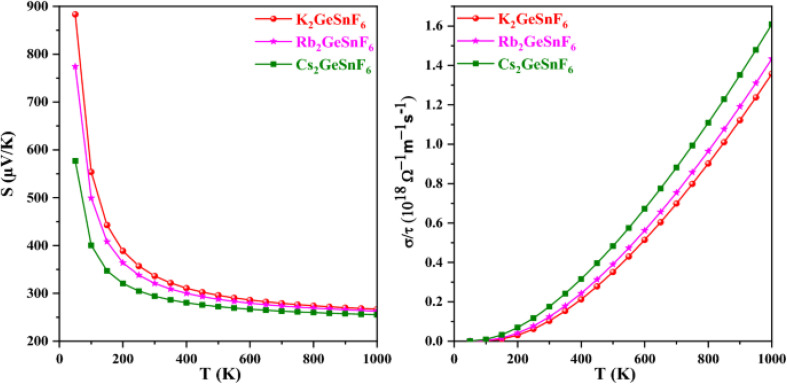
Variation of electrical conductivity and see beck coefficient with temperature for A_2_GeSnF_6_(A = K, Rb, Cs).^210^

In the context of thermoelectric properties, perovskites are advantageous for converting surplus heat into electrical energy due to their affordability, high electrical conductivity, and environmental friendliness. The BoltzTraP code is employed to compute key thermoelectric parameters, including electrical conductivity, Seebeck coefficient, and figure of merit, using structural and electronic data from the Wien2k code. For A_2_GeSnF_6_ (A = K, Rb, Cs), these materials exhibit promising thermoelectric properties. Fig. 13 shows the variation of electrical conductivity with temperature, demonstrating that as the temperature increases, charge carriers move more easily from the valence band (VB) to the conduction band (CB), validating the semiconducting properties of these materials. The electrical conductivity decreases as Cs is replaced by Rb and K, reflecting the impact of atomic size and Coulomb repulsion. The increase in electrical conductivity with temperature indicates the negative temperature coefficient of resistance, a hallmark of semiconducting materials.

#### 3.5.2 Battery and supercapacitor integration

Beyond energy generation, MHPs are also being explored for their potential in energy storage systems, particularly in next-generation batteries and supercapacitors. The high ionic conductivity of specific perovskite materials makes them strong candidates for use as electrolytes or active materials in these devices. In particular, the incorporation of MHPs into lithium-ion or sodium-ion battery systems could significantly improve the energy density and charge/discharge rates, addressing some of the critical challenges currently faced by traditional energy storage solutions. Furthermore, MHPs' high ionic conductivity can also be utilized in supercapacitors, which require fast ion transport to achieve high power densities and rapid charge/discharge cycles. By optimizing the structural and electrochemical properties of MHPs, it is possible to develop advanced hybrid energy storage systems that combine the best features of both batteries and supercapacitors, leading to efficient and long-lasting energy storage solutions for a range of applications, from portable electronics to grid-scale energy storage.

Jung Hwan *et al.*^211^ provide a comprehensive overview of the progress in light-material interactions (LMIs), focusing on laser and flashlight sources for energy conversion and storage applications. This review highlights key LMI parameters, such as light sources, interaction time, and fluence, to emphasize their role in material processing. It covers a range of light-induced photothermal and photochemical processes, including melting, crystallization, ablation, doping, and synthesis, which are critical for developing energy materials and devices. The study also discusses various energy conversion and storage applications enabled by LMI technologies, such as energy harvesters, sensors, capacitors, and batteries. It outlines the challenges associated with LMIs, including the complexity of the mechanisms and the high degrees of freedom involved in the interactions. Despite these challenges, the authors argue that advancements in optical technologies, driven by thorough academic research and multidisciplinary collaborations, offer substantial potential for future energy systems.

**Fig. 14:**
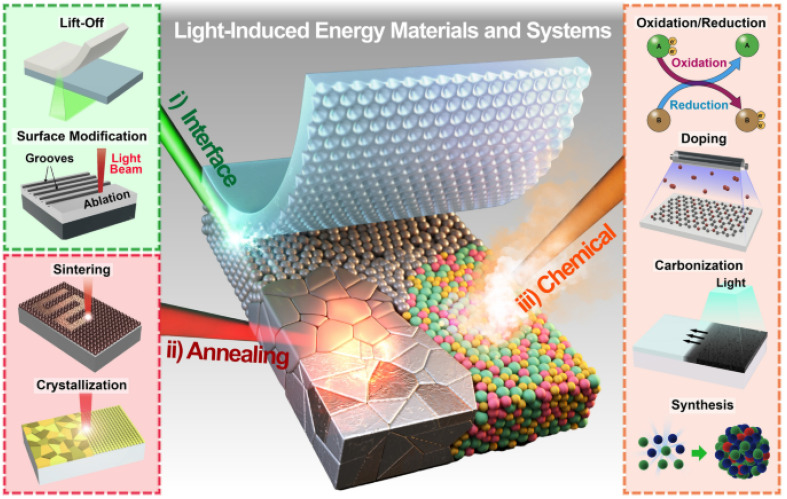
Schematic of the overall concept of light-induced energy materials and devices.^211^

**Fig. 15:**
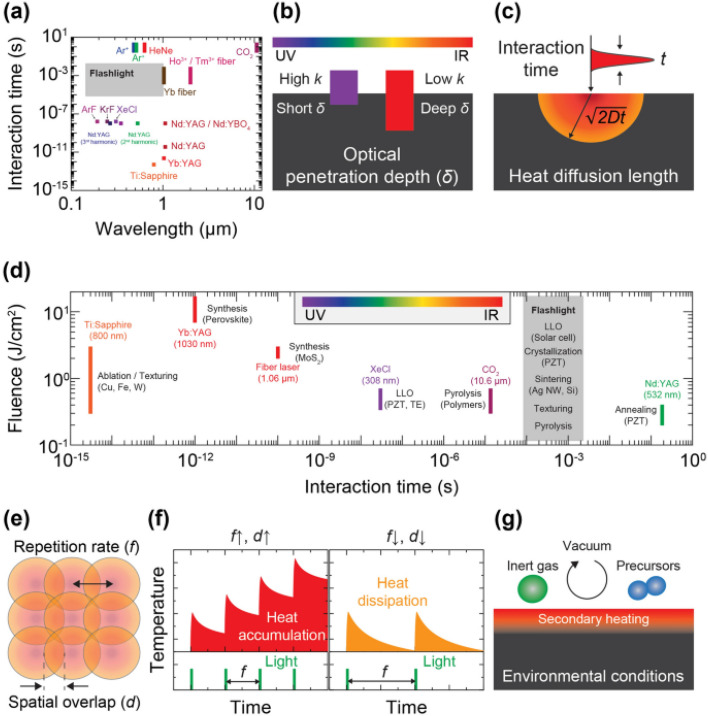
(a) Wavelength and interaction time features of lasers and flash lamps; (b) light wavelength influencing the optical penetration depth for light-absorbing materials; (c) interaction time that determines the heat diffusion length; (d) fluence and interaction time regime related to LMI events, (e) repetition rate and spatial overlap, contributing to (f) heat accumulation/dissipation effects; (g) environmental conditions that trigger physicochemical reactions.^211^

The review introduces the concept of light-induced energy materials and devices and discusses how LMIs can precisely and selectively manipulate thermal energy transport within controlled time intervals. This level of control is difficult to achieve using traditional microfabrication and furnace-based annealing methods. However, the intricate nature of LMIs requires careful consideration of multiple simultaneous parameters to achieve the desired physicochemical reactions. Fig. 14 presents a schematic of the overall concept of light-induced energy materials and devices. In addition, Fig. 15 illustrates key factors that contribute to the interactions between light and materials, including incident wavelength, irradiation duration, fluence or power, repetition rate, spatial overlap, and environmental conditions. These parameters must be optimized to achieve precise control over the photonic effects in materials, which is essential for the development of energy materials and devices.^211^

#### 3.5.3 Perovskite-based sensors and optoelectronics

The unique tunability of MHPs' optoelectronic properties opens exciting possibilities in the fields of sensors and optoelectronics. MHPs can be engineered to absorb or emit light across a wide range of wavelengths, which makes them highly versatile for use in applications such as photodetectors, light-emitting diodes, and flexible electronics. The ability to adjust the bandgap of MHPs by altering their composition allows them to be tailored for specific wavelengths, making them ideal for use in sensors that require high sensitivity across different environmental conditions. Additionally, MHPs are being incorporated into the development of smart devices, particularly in the context of the Internet of Things (IoT), where low-power, high-performance sensors are needed for a wide array of applications, from environmental monitoring to wearable technology. Flexible electronics based on MHPs are another area of significant interest, as they offer the potential for lightweight, durable, and highly efficient devices that can be integrated into new form factors, such as stretchable and bendable sensors for health monitoring, smart textiles, and wearable devices.

Mohamed *et al.*^212^ report the study of the chemical and physical characteristics of all-inorganic metal halide perovskites CsNBr_3_ (N^2+^ = Ge, Sn, Pb) through first-principles approaches using density functional theory. Three different DFT approximations, Perdew–Burke–Ernzerhof (PBE), PBESOL, and Wu–Cohen (WC), within the generalized gradient approximation (GGA) are employed, along with the Kohn–Sham (KS) equation as executed in the WIEN2k package. To reproduce accurate energy gaps (*E*_g_) in the PBE band structures of CsNBr_3_ perovskites, the hybrid functional HSE06 is used. The results from the GGA approaches for the structural, electronic, and optical properties are consistent with experimental data and previous DFT calculations, with the PBE method yielding values closest to experimental findings. The study reveals that CsNBr_3_ perovskites exhibit nonmagnetic and semiconducting properties, with reliable *E*_g_ values localized at the *R*-symmetry point. Additionally, the photonic energy-dependent optical properties, including the real and imaginary parts of the dielectric function, conductivity, reflectivity, refractive index, absorption, and extinction coefficients, are calculated using the GGA approaches.

**Fig. 16:**
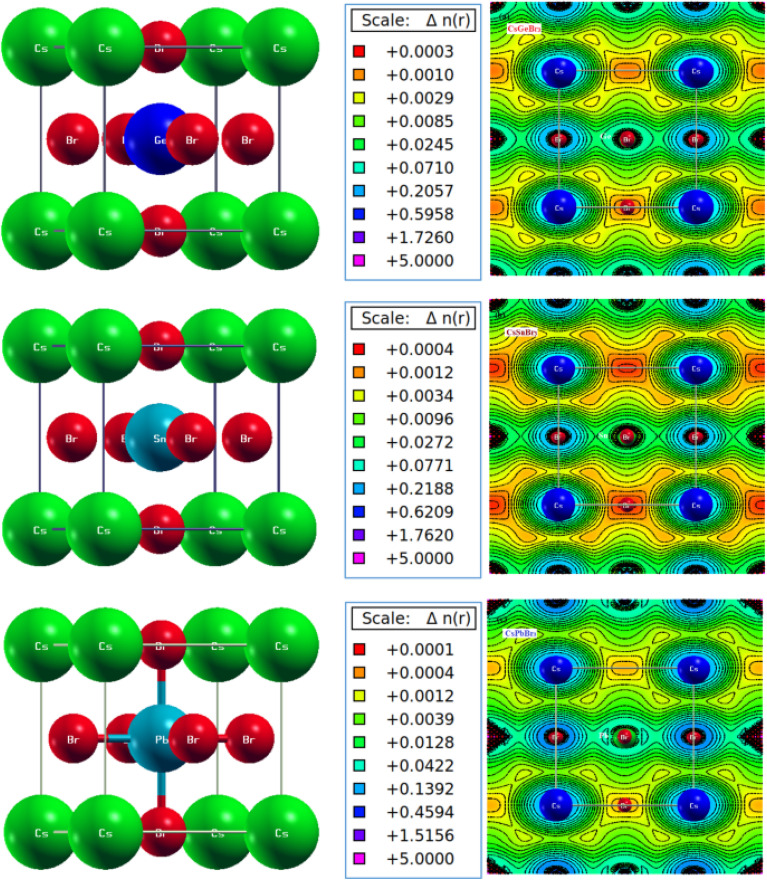
The crystal structure and electronic charge density in (100) plane of the unit cells for metal halide perovskites CsNBr_3_ (N^2+^ = Ge, Sn, Pb), in cubic symmetry (space group *Pm*3̄*m*; IT No. 221) optimized using GGA approaches. Color legend: cesium (green), germanium (dark Blue), tin (sky blue) and lead (steel blue), and bromine (red).^212^

The semiconducting direct (*E*_g_ = 0.9814–1.9086 eV) and high optical absorption suggest that the CsNBr_3_ perovskites are promising candidates for designing inorganic photovoltaic (PV) solar cells, photodetectors, photodiodes, and other PV devices operating in the ultraviolet-visible range. Fig. 16 displays the crystal structure and electronic charge density in the (100) plane of the unit cells for the metal halide perovskites CsNBr_3_ (N^2+^ = Ge, Sn, Pb) in cubic symmetry (space group *Pm*3̄*m*; IT No. 221), optimized using GGA approaches. Paul Hänsch *et al.*^213^ demonstrated that their oxygen sensor has a detection limit of 70 ppm and a response time of 400 ms, which is considered relatively slow. The enhanced conductivity of MAPbI_3_ is attributed to the filling of iodide vacancies by physiosorbed oxygen. This mechanism was further validated by exposing the device to methyl-iodide vapor, which reduced the oxygen sensing performance by decreasing the available iodide vacancies. Additionally, the authors emphasized that the fabrication of the perovskite films plays a significant role in the sensor's effectiveness, as these vacancies tend to be concentrated at the surface and grain boundaries.

**Fig. 17:**
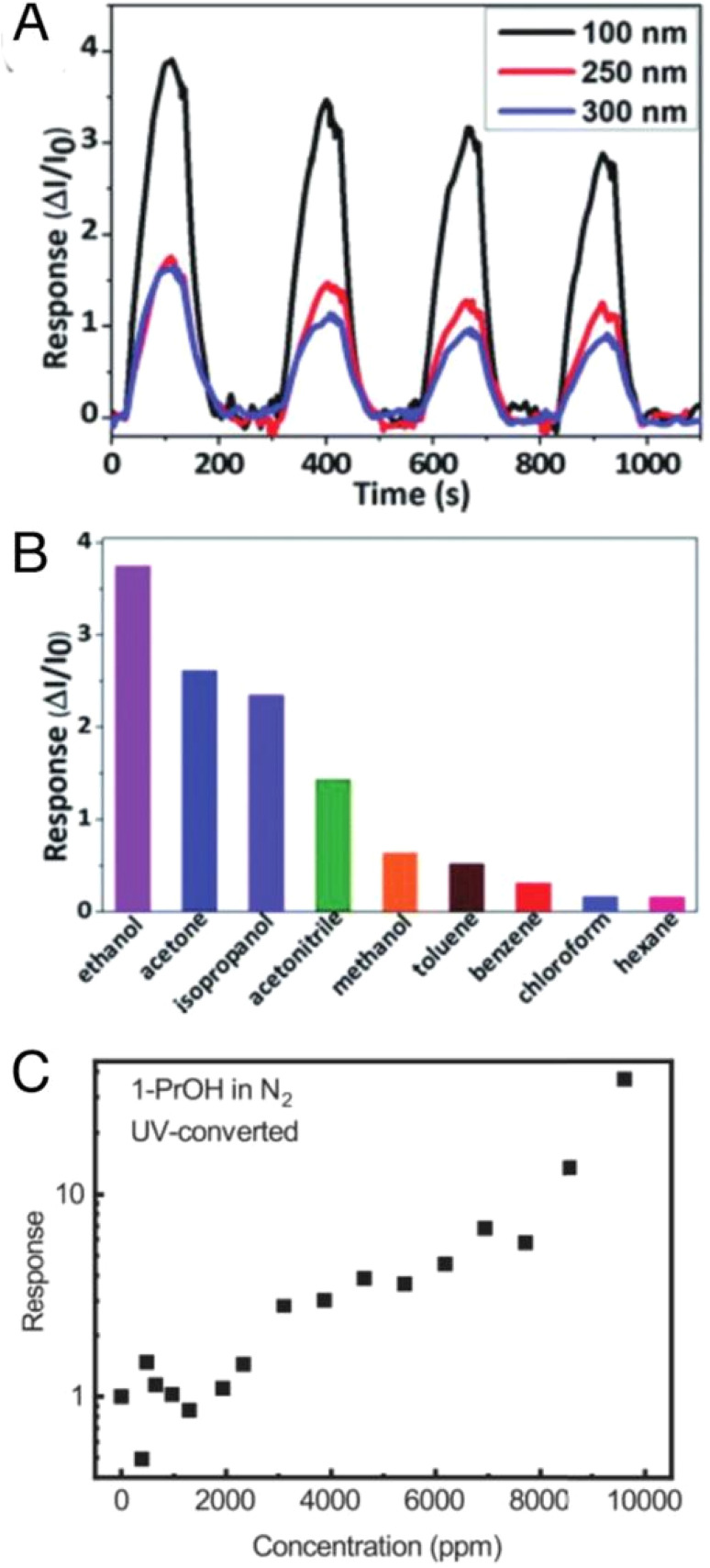
(A) Gas sensor response to ethanol depending on the layer thickness, (B) screening for different organic solvents, and (C) gas sensor responses from Groeneveld and Loi16 of 1-propanol detection.^213^

Following these initial findings, research shifted towards using metal halide perovskites for detecting volatile organic compounds (VOCs). In 2020, MAPbI_3_ was demonstrated to be effective in detecting ethanol. Paul Hänsch *et al.*^213^ examined the ethanol sensing capabilities and observed the effect of different film thicknesses (Fig. 17). They found that thinner films lead to a higher current response, suggesting that the interaction between the analyte and the surface of the material determines the detection. MAPbI_3_ showed a slight preference for ethanol over acetone, isopropanol, and other VOCs. Their most effective sensor had a limit of detection (LOD) of 1300 ppm, a response value of 3.7 at 10 000 ppm and 1 V, and a response time of 66 seconds.

The oxygen sensor also had a detection limit of 70 ppm and a slow response time of 400 ms. As with ethanol detection, the increased conductivity of MAPbI_3_ is explained by the physisorption of oxygen into iodide vacancies. The exposure to methyl-iodide vapor also demonstrated the trap healing mechanism, where fewer iodide vacancies lead to reduced sensing capabilities. The study further showed that the film's fabrication significantly impacted the sensor's performance, as vacancies were primarily located at the surfaces and grain boundaries.

In VOC detection, MAPbI_3_ exhibited slight selectivity for ethanol compared to acetone, isopropanol, and other VOCs. Thinner films again led to a higher current response, confirming that surface interactions are key in detection. Their best device had a detection limit of 1300 ppm, a response of 3.7 (at 10 000 ppm and 1 V), and a response time of 66 seconds. Fig. 17 illustrates the gas sensor responses to ethanol based on layer thickness [Fig. 17(A)], VOC screening [Fig. 17(B)], and 1-propanol detection [Fig. 17(C)].

Gas and VOC sensing is crucial in industries like food, agriculture, and healthcare. Electronic noses must be sensitive, selective, reliable, and inexpensive, but it is rare to meet all these demands for every analyte. Current technologies mainly rely on metal oxide semiconductors, which have limitations such as poor selectivity and the need for high temperatures to improve sensitivity. This has led to the exploration of alternative material platforms like metal halide perovskites, which show exceptional sensitivity to environmental changes. The first demonstration of gas sensing with metal halide perovskites at room temperature occurred in 2016, and recent reports highlight their significant potential for detecting various gases and VOCs. This paper will summarize these developments, discuss the mechanisms underlying gas sensing, and explore the prospects for highly sensitive and selective sensors based on metal halide perovskites.

## 4. Challenges and future perspectives

The potential of metal halide perovskites in energy applications, particularly in solar cells, light-emitting devices, and lasers, is undeniable; however, several significant challenges must be addressed before these materials can achieve widespread commercial viability. One of the most pressing concerns is stability, as perovskite-based devices tend to degrade when exposed to environmental factors such as moisture, heat, and oxygen, which negatively impact their performance and longevity. Enhancing the environmental stability of these materials through encapsulation, surface passivation, and the development of all-inorganic variants is essential for long-term reliability. Another key challenge is the toxicity of lead in perovskite materials, which raises environmental and health concerns, particularly in the event of device failure. To mitigate this, research into lead-free alternatives, such as tin-based perovskites, and the adoption of recycling strategies for lead recovery from decommissioned devices is critical for sustainability. Furthermore, scaling up perovskite fabrication from lab-scale to large-area modules presents challenges related to uniformity, reproducibility, and efficiency. To address this, scalable deposition methods like roll-to-roll printing and blade coating must be refined for mass production, alongside machine learning techniques to optimize manufacturing processes. Additionally, the charge transport properties of perovskite materials, often hindered by defects, need to be improved. Defect passivation techniques, such as organic and inorganic passivators and interface engineering, are being explored to enhance charge mobility and reduce recombination losses, which can improve the overall efficiency of perovskite-based devices. While perovskite solar cells have achieved impressive power conversion efficiencies (PCEs), they still lag behind silicon-based cells in terms of stability and consistency. Future efforts will focus on developing tandem architectures, stacking perovskites with other semiconductors to maximize light absorption and energy conversion. Additionally, the development of energy-efficient perovskite LEDs and lasers will benefit from advancements in charge injection layers, light extraction, and interface optimization. The commercialization of perovskite-based technologies also faces economic and regulatory challenges; the cost-effectiveness of large-scale production must compete with existing technologies, and regulatory hurdles must be overcome, particularly regarding lead toxicity. However, as scalable manufacturing improves and regulatory frameworks evolve, perovskite-based energy devices could revolutionize the renewable energy sector, offering a sustainable, cost-effective solution to global energy demands. By overcoming these challenges through continued innovation, metal halide perovskites could play a pivotal role in the transition to more efficient, environmentally friendly, and affordable energy solutions in the future.

## 5. Conclusions

In conclusion, metal halide perovskites represent a groundbreaking class of materials with immense potential for revolutionizing energy applications, particularly in solar cells, light-emitting devices, and lasers. Recent advancements have shown remarkable progress in improving their efficiency, versatility, and performance, making them strong contenders for next-generation energy solutions. However, several challenges remain, including long-term stability, toxicity, and scalability, which must be addressed before MHPs can be widely adopted in commercial products. The development of lead-free alternatives, enhanced encapsulation, and defect passivation techniques will be crucial in overcoming these barriers. Furthermore, scalable fabrication methods, coupled with advances in machine learning and high-throughput screening, hold promise for accelerating the discovery of more efficient and stable perovskite compositions while enabling cost-effective mass production. The continued integration of *in situ* characterization and *operando* studies will deepen our understanding of degradation mechanisms, allowing for the design of more robust devices. Despite the challenges, the potential of MHPs to transform the energy sector remains immense, with ongoing research pointing toward a future where perovskite-based technologies contribute significantly to sustainable energy solutions. With concerted efforts across materials science, engineering, and environmental sustainability, MHPs are poised to play a key role in the global transition to clean, renewable energy.

## Author contributions

Sonia Soltani: conceptualization, supervision, manuscript writing—original draft, and final editing. Mokhtar Hjiri: literature review, data analysis, writing—review and editing. Najwa Idris A. Ahmed: investigation, visualization, and support in manuscript drafting. Anouar Jbeli: data collection, figures preparation, and technical validation. Abdullah M. Aldukhayel: literature curation, critical revisions, and methodology support. Nouf Ahmed Althumairi: writing—review and editing and contributing to future perspectives section. All authors have read and approved the final version of the manuscript.

## Conflicts of interest

The authors declare that they have no conflict of interest related to this work.

## Data Availability

No primary research results, software or code have been included and no new data were generated or analysed as part of this review.
